# Infection control in general practices in Buffalo City and OR Tambo District Municipalities, South Africa

**DOI:** 10.4102/phcfm.v4i1.268

**Published:** 2012-02-14

**Authors:** Don O'Mahony

**Affiliations:** 1 Department of Family Medicine, Walter Sisulu University, South Africa

## Abstract

**Background:**

Good infection control practices are effective in reducing rates of infection in health care settings. Studies in primary care in developed countries indicate that many general practitioners (GPs) do not comply with optimal infection control practices. There are no published studies from developing countries in Southern Africa.

**Objectives:**

The aim of this study was to describe infection control practices in private GP surgeries in the Buffalo City and OR Tambo District Municipalities in the Eastern Cape Province, South Africa.

**Method:**

A literature review was conducted to appraise current best practice with respect to Standard Infection Control and Transmission Based Precautions. A questionnaire, inquiring into GPs’ actual practices, was posted to each surgery.

**Results:**

The valid response rate was 34% (47/140). Methods used to sterilise instruments in 40 practices were: ultraviolet sterilisation (23), chemical disinfection (14), boiling water (7), and steam autoclave (2). Compounds used for chemical disinfection included organotin quaternary, chlorhexidine and benzyl ammonium chloride with a quaternary complex. Twenty-two (47%) used a hand rub. Sixteen (35%) GPs stated that they had a policy to promptly triage patients who are coughing, and 23 (50%) had a policy for airflow movement in the surgery. All practices appropriately disposed of sharps. Thirty-seven (80%) expressed interest in a seminar on infection control.

**Conclusions:**

Overall, GPs were aware of infection control precautions. Ultraviolet sterilisers and chlorhexidine are not recommended, however, for sterilisation or high level disinfection of medical instruments, and their use should be discontinued. Hand rubs are underutilised. GPs should implement Transmission Based Precautions to prevent airborne and droplet infections.

## Abstrait

**Contrôle des infections dans les cabinets de médecine générale dans les municipalités de Buffalo City et du district d'OR Tambo, en Afrique du Sud**

**Contexte:**

De bonnes pratiques de contrôle des infections sont efficaces pour réduire les taux d'infection dans les lieux administrant des soins de santé. Des études sur les soins primaires dans les pays développés indiquent que de nombreux médecins généralistes ne respectent pas les pratiques de contrôle des infections optimales. Aucune étude émanant des pays en voie de développement en Afrique australe n'a jamais été publiée.

**Objectifs:**

L'objectif de cette étude consistait à décrire les pratiques de contrôle des infections dans les cabinets de consultation de généralistes privés dans les municipalités de Buffalo City et du district de Tambo OR, dans la Province du Cap oriental, en Afrique du Sud.

**Méthode:**

Une analyse bibliographique a été réalisée afin d’évaluer la meilleure pratique actuelle quant aux précautions standards pour contrôler des infections et celles fondées sur la transmission. Un questionnaire, s'intéressant aux pratiques réelles des généralistes, a été affiché dans chaque cabinet.

**Résultats:**

Le taux des réponses valides était de 34% (47/140). Les méthodes utilisées pour stériliser les instruments dans 40 cabinets étaient les suivantes: stérilisation par ultraviolet (23), désinfection chimique (14), eau bouillante (7) et autoclave à vapeur. Les composés utilisés pour la désinfection chimique incluaient le composé quaternaire organostannique, la chlorhexidine et le chlorure de benzylammonium quaternaire. Vingt-deux généralistes (47%) utilisaient un désinfectant des mains. Seize (35%) ont affirmé appliquer une politique permettant de trier rapidement les patients qui toussaient, et 23 (50%) une politique relative à la ventilation dans le cabinet. Tous les cabinets jetaient correctement les aiguilles usagées. Trente-sept (80%) ont indiqué être intéressés par un séminaire sur le contrôle des infections.

**Conclusion:**

Dans l'ensemble, les généralistes connaissaient les précautions permettant de contrôler les infections. Les stérilisateurs à ultraviolet et la chlorhexidine ne sont cependant pas recommandés pour la stérilisation ou la désinfection de haut niveau des instruments médicaux, et leur utilisation doit être abandonnée. Les désinfectants des mains sont sous-utilisés. Les généralistes devraient mettre en pratique les Précautions fondées sur la transmission afin de prévenir les infections transmises par voie aérienne et par gouttelettes.

## Introduction

### Setting

Good infection control practices are effective in reducing rates of infection in health care settings.^1^ Furthermore, health professionals have ethical and legal obligations to safeguard their patients and staff.^2^ Studies from primary care in developed countries indicate that many general practitioners (GPs) do not comply with standard infection control practices.^3, 4, 5, 6, 7, 8^

In a postal questionnaire survey of general practices in Northern Ireland:

- only 51% of those who had a desktop steriliser had written instructions for its operation
- no steriliser was cleaned daily
- no practice changed the steriliser water on a daily basis.^3^

In a survey of all 92 general practices in a Health Board area in the UK, it was reported that:

- only 33% practices had an alcohol hand rub
- there was a non-availability of personal protective equipment such as eye protection (81%) and face masks (62%)
- resheathing of needles was carried out by 55%
- 70% performed no user check at all on their autoclaves
- 15% of instruments were inadequately decontaminated
- 38% had unsatisfactory methods of treating blood spillages.^5^

In a postal questionnaire study of a random sample of 200 Irish GPs, 2% disposed of sharps in the rubbish bin and 6% placed their sharp containers with the domestic refuse.^8^ There are no published studies or guidelines on infection control in general practice in South Africa. The aim of this study is to describe the infection control practices in the surgeries of GPs in private practice in two municipalities in the Eastern Cape Province of South Africa.

#### Research significance

The study is the first to document infection control practices in private general practices in South Africa. Ultraviolet sterilisers and chlorhexidine were used for the sterilisation of instruments that enter tissues. However, these methods are not recommended for sterilisation or high level disinfection and their use should be discontinued. Hand rubs were underutilised. Transmission Based Precautions were implemented by a minority. General Practitioners expressed a need for infection control education.

## Ethical considerations

Ethical approval was obtained from the Human Research Committee at the Faculty of Health Sciences, Walter Sisulu University (No. 0017/009).

## Method

### Design

The author conducted a literature search in English-language publications to appraise current best practice in infection control in primary care in order to establish a 'gold standard’ with which to compare general practices in South Africa. Many relevant guidelines and policies were found for infection control from agencies in different countries.^9, 10, 11, 12, 13, 14^ The recommendations were broadly similar. However, only one set of guidelines provided details of using a systematic review process.^14^ The guidelines from the Infection Control Team of Health Protection Scotland, a division of NHS National Services Scotland, are updated annually after a literature review and are readily accessible on their website.^14^ Identified articles are reviewed in accordance with a model for critical appraisal of scientific studies,^15^ Scottish Intercollegiate Guidelines Network (SIGN 50) methodology for systematic review and meta-analyses,^16^ and the Appraisal of Guidelines for Research and Evaluation (AGREE) instrument for the evaluation of guidance documents as appropriate.^17^ The author did not consider it necessary to review the original articles and accepted the recommendations, because they followed a rigorous review process or were the result of expert consensus.

### Setting

The study population comprised GPs in private practice in the OR Tambo District Municipality and Buffalo City Municipality. They were identified by:

- checking the website of the Board of Healthcare Funders of South Africa, because GPs must register with the Board to receive payments from Medical Aid Societies
- contacting the Independent Practitioner Organizations that are based in the Municipalities
- the list of GPs that take elective students from Walter Sisulu University
- phone calls to one or more GPs in each town to identify colleagues. Each practice was phoned to confirm the postal address and the identity of partners, if any.

### Materials

A questionnaire was designed to assess seven (relevant to general practice) of the nine elements of Standard Infection Control Precautions,^14^ which are based on Garner's criteria.^18^ These were, hand hygiene, personal protective equipment, prevention of occupational exposure to infection, management of blood and body fluid spillages, management of patient care equipment, safe disposal of waste including sharps, and safe management of linen. Also assessed were two of the three elements of Transmission Based Precautions (Additional Precautions),^9^ namely airborne and droplet precautions. To assess management of patient care equipment, items were selected that needed sterilisation and high level disinfection, after Spaulding's classification.^19^ All instruments that enter tissues are classified as critical, for example forceps and scalpel, and require sterilisation. Instruments that have contact with mucous membranes or non-intact skin but do not enter tissues are classified as semi-critical, for example vaginal and ear speculae, and require high level disinfection. A pilot questionnaire was completed by five GPs outside the study areas and ambiguities were corrected. One study questionnaire, together with a covering letter and reply-paid envelope, was posted to each practice, irrespective of the number of partners, in September 2009 with the request that a GP or practice manager completes it. A follow-up questionnaire was posted 3 months later to non-respondents.

## Results

One hundred and forty practices were identified, comprising 171 GPs. From the two postings of questionnaires, 50 were returned. Of those returned, three GPs stated they had retired and thus were excluded from analysis. The valid response rate was 34% (47/140). Of the responders, 36 were male and 11 were female. The numbers per age group were: 12 (30–39 years); 10 (40–49 years); 17 (50–59 years); six (≥ 60 years); and 2 did not state their age. Thirty-three were in solo practice and fourteen in partnerships. Of those in partnerships, 13 were in groups of two to three doctors and one was in a partnership of seven. Eighteen (38%) practices employed one or more registered nurses. Twenty-eight (60%) GP practices were in the Buffalo City Municipality and 19 (40%) in the OR Tambo District Municipality.

**Hand Hygiene**: Of 138 consulting or treatment rooms in the 47 practices, 109 (79%) had hand basins and 23 (21%) basins had elbow operated taps. For drying hands, GPs used one or more of the following: cloth on a rail 35 (74%), paper towels 30 (64%), or cloth rolls 11 (23%). One used a hot air dryer. Twenty-two (47%) used a hand rub. A hand rub was defined as an antiseptic and/or alcohol based preparation to decontaminate the hands without the need for water and which requires no rinsing or drying with towels or other devices. Of those using a hand rub, one used methylated spirit and two an unspecified alcohol.

**Personal Protective Equipment**: Of 41 respondents who did minor surgery (procedures that could expose them to splashes of blood or body fluids), (Table 1), 24 (59%) used a mask, 15 (37%) used a visor, five (12%) wore boots or shoe covers and 27 (66%) wore an apron.

**TABLE 1:** Procedures carried out at General Practitioner's practices (*N* = 47).

Procedure	*n*	%	Procedure	*n*	%
Ear syringing	40	85	IUCD‡	12	26
Incision of abscesses	38	81	Implants§	9	19
Dressing of wounds	38	81	Proctoscopy	6	13
Suturing	34	72	ROC¶	5	11
Pap smear	34	72	TOP††	4	9
Biopsies	22	47	Gastroscopy	1	2
Circumcision	19	40	Deliveries‡‡	1	2
Drainage†	15	32	Other	0	-

*Source*: Authors original data

*N*, The total number of patients; *n*, Given as a means of number.

† Drainage of body cavity fluids (pleural, abdominal).

‡ IUCD (Intra-uterine contraceptive device).

§ Contraceptive implant.

¶ Instrumental removal of retained products of contraception.

†† Termination of pregnancy by manual vacuum aspiration or suction curettage.

‡‡ Deliveries on surgery premises.

**Immunity to Hepatitis B Virus (HBV):** Thirty-four (72%) knew they were immune to HBV infection and seven (15%) knew that all their staff was immune.

**Management of blood and body fluid spillages (excluding faeces and urine):** Forty (85%) stated that they applied disinfectant onto spillages, five (11%) did not and two did not answer. Some used more than one type of disinfectant. The disinfectants applied, with the trade names in brackets, and the number of respondents, are: chlorhexidine and cetrimide (Savlon^®^) nine; chlorhexidine (Hibitane^®^, Biotane^®^) seven; sodium hypochlorite (Jik^®^, Domestos^®^) six; chlorine (Biocide^®^) two; ethyl alcohol with methyl alcohol (Surgical Spirit) two; chloroxylenol (Dettol^®^) two; 4-chloro-M-Cresol (Jeyes Fluid^®^) one; and organotin quaternary (SteriTech^®^) one. Thirteen did not specify the disinfectant used.

**Disinfection Methods for selected medical devices:** Various methods were used to disinfect selected medical devices in GP practices (Table 2).

**TABLE 2:** Methods used to disinfect selected medical devices in General Practitioner's practices (*N* = 47).

Device	Recommended Methods^20^	Methods Used by GP Practices†					

		General Practices		Ultraviolet Steriliser	Chemical Disinfectant	Boiling Water	Steam Steriliser

		*n*	%				
Forceps	Steam steriliser	39	83	22	14	6	2
Scalpel	Steam steriliser	40	85	23	14	7	2
Vaginal speculum	Steam steriliser Boiling water‡	36	77	20	14	9	2
Ear syringe	Steam steriliser Boiling water‡	33	70	16	15	5	-
Auroscope speculum§	Steam steriliser Boiling water‡	43	91	12	22	6	2
Gastroscope	Chemical	1	2	1	1	-	-
Proctoscope	Steam steriliser Boiling water‡	5	11	5	2	2	1

*Source*: Authors original data

*N*, The total number of patients; *n*, Given as a means of number.

† Devices were disinfected by more than one method.

‡ Whilst a steam steriliser is optimal, immersion in boiling water for ≥ 20 minutes is acceptable for high-level disinfection.^21^

§ For auroscope speculums, there were seven respondents who did not detail the disinfection method.

**Boiling**: Of the seven who used boiling water for disinfection of scalpel handles, five (71%) boiled for (< 20) minutes and two (29%) for (≥ 20) minutes. Of the nine respondents who used boiling for disinfection of vaginal speculae, six (67%) boiled for (< 20) minutes and three (33%) for (≥ 20) minutes.

**Steam Steriliser (Autoclave)**: Two GPs used autoclaves: one used settings of 114 °C at 220 kPa (2.2 bar) for 30 minutes and one used 100 °C at 200 kPa (2 bar) for 30 minutes.

**Chemica**l **Disinfection**: The chemicals used for sterilisation in practices (number of practices) were organotin quaternary (7), chlorhexidine and cetrimide (3), chlorhexidine (2) and benzyl ammonium chloride with a quaternary complex (Instrubac^®^) (1). For high level disinfection, the chemicals (number of practices) were organotin quaternary (6), chlorhexidine and cetrimide (4), chlorhexidine (2), benzyl ammonium chloride with a quaternary complex (1) and alcohol wipe (1). Not all GPs provided details of chemical disinfection. For example, only 14 (64%) of the 22 who used a chemical for cleaning auroscope speculae specified the agent.

**Disposable instruments:** Five GPs used disposable vaginal speculae and four used disposable auroscope speculae.

**Waste management**: Forty-five (96%) GPs separated ‘risk waste’ from ‘general waste’. Risk or healthcare waste was defined as posing a risk of infection, for example blood, body fluids and tissue. For risk waste, 26 (55%) used bins with pedal operated lids and 40 (85%) used bins lined with plastic bags. For disposal, 38 (81%) used a registered medical waste disposal company, 5 (11%) used a hospital incinerator and 6 (13%) used the municipal domestic waste disposal service.

**Disposal of Sharps**: Forty-one (87%) GPs used containers compliant with South African National Standards (SANS) for disposal of sharps. Eleven (23%) used empty medicine containers either alone or combined with SANS approved containers. For disposal of containers, 41 (87%) used a medical waste disposal company and 6 (13%) used a hospital incinerator.

**Peak Flow Meter**: Twenty-six (55%) GPs used a Peak Flow Meter. Of these, 11 (42%) used a disposable mouthpiece. Of the 15 that reused mouthpieces, 10 cleaned them as follows: 3 with chlorhexidine and cetrimide, 3 with chlorhexidine, and 1 each with chlorine (Milton^®^), an alcohol swab, water and organotin quaternary. Five did not specify how they cleaned them.

**ECG**: Of 25 GPs who performed ECGs, 14 (56%) used disposable electrodes. Of the 11 who used reusable electrodes, 6 specified how they disinfected them; 4 used povidone-iodine or alcohol base, and 2 wiped with water.

**Ultrasound scan**: Twenty-seven (57%) used an ultrasound scanner and sixteen specified how they cleaned the probe between patients: six used alcohol, one used chlorhexidine and cetrimide, one used soap and water, six wiped with paper towels and two used water.

**Airborne and droplet precautions**: Sixteen (34%) GPs stated that they had a verbal or written policy to promptly triage patients who are coughing, from the waiting room into an examination room or into a separated well-ventilated waiting area. Twenty-three (50%) stated that they had a policy for ventilation in the surgery in order to reduce exposure to respiratory pathogens.

**Safe management of linen**: Forty-one (87%) GPs laundered soiled linen at the surgery or at home, three at commercial laundries and two at unspecified sites. For washing by machine and/or manually, 30 used hot water and 15 used cold water. Only four respondents detailed the operating temperature of the washing machine; three specified 40 °C and one specified 30 °C. Only one respondent used disposable linen.

**Interest in a seminar**: Thirty-seven (78%) expressed interest in a seminar on infection control in general practice.

**Comments**: Twenty-two GPs made comments or suggestions.

The main themes were:

- four requested guidelines on infection control
- three stated that the questionnaire was an eye opener or showed a need to improve on infection control
- two said that it was valuable research on an important topic
- two stated that they do very few procedures because there are government or private health facilities nearby
- two believed that they have always maintained a good level of infection control.

## Discussion

The GPs in this study were cognisant of infection control in their practices, but although they took precautions, some areas for improvement were identified. It is more likely that GPs will disinfect their hands after every patient encounter if there is a hand basin in each consulting and treatment room, and if they use a hand rub. Only 79% of rooms had basins and more than half of the GPs did not use a hand rub. This compares with a UK study where only 33% of practices had a handrub.^5^ Alcohol-based hand rubs remove organisms more efficiently; require less time; irritate skin less often than hand washing with soap or other antiseptic agents and water; and are associated with better compliance.^22^ Some GPs used methylated spirit and alcohol as a hand rub. These are sub-optimal because alcohol does not easily denature microbial proteins in the absence of water.^23^ Hand rubs containing 60% – 80% alcohol are most effective as antiseptics.^23^ When hands are visibly dirty or contaminated with proteinaceous material or are visibly soiled with blood or other body fluids, it is recommended that hands are washed with either a non-antimicrobial soap and water or an antimicrobial soap and water. It is not essential to have soap dispensers because the actual hazard of transmitting microorganisms through hand washing with previously used soap bars is negligible.^22^ With regard to the washing of hands, elbow operated taps reduce the risk of cross-contamination.^24^ Only 21% of basins in the GP surgeries had elbow operated taps. Seventy-four per cent of GPs used a hand towel on a rail for drying hands. Reusing or sharing towels should be avoided because of the risk of cross infection.^25^ There seems to be little difference between individual paper towels and (hot) air dryers in reducing pathogens on washed hands.^25, 26^ However, paper towels may be preferred because air drying takes longer to dry hands and may cause aerosolisation of pathogens leading to cross infection.^27^

Forty-one per cent of GPs did not use a mask and 63% did not use a visor to protect themselves from splashes when performing risk procedures. Furthermore, more than a quarter of GPs did not know their own HBV immune status and 85% did not know the status of their staff. GPs should be aware of the risks of HBV infection and if they chose not to immunise themselves, they should be aware of risks to their staff. Whilst percutaneous injury is the most efficient mode of HBV transmission, there is no recollection of needle prick in most cases of HBV infection in health-care staff (with no history of non-occupational exposure).^28, 29^ HBV can survive in dried blood on environmental surfaces for up to 1 week.^30^ It is hypothesised that HBV infection results from direct or indirect exposure to blood and body fluids onto damaged skin or onto mucosae.^31^ Thus, all categories of staff that access the consultation and treatment rooms are at risk of HBV infection and should be offered testing, and immunisation if non-immune.^32^

A minority of those who reported that they use a disinfectant on spillages of blood and body fluids, made use of sodium hypochlorite. Sodium hypochlorite and sodium dichloroisocyanurate are the recommended agents.^33^ Chlorhexidine was commonly used but is not recommended.^33^ It is an antiseptic that should be used only on skin and wounds. Whilst it has good activity against Gram-positive bacteria, it has less activity against Gram-negative bacteria and fungi, and minimal activity against mycobacteria and non-enveloped viruses like rotavirus, adenovirus and enteroviruses.^22^ Furthermore, it can be contaminated readily (particularly with *Pseudomonas aeruginosa)* after decanting or diluting.^20^

With regard to sterilisation and disinfection of items of patient care equipment, the most common methods used by GPs were ultraviolet sterilisers, chemical disinfection, boiling water and steam (autoclave). Ultraviolet radiation (UV) at certain wavelengths is germicidal but its effectiveness is limited *inter alia* by organic matter, wavelength, temperature, the type of microorganism and UV intensity, which is affected by distance and dirty fluorescent tubes.^34^ UV has limited energy and does not penetrate dust and mucous with the result that any residue on instruments may not be sterilised. It must be kept in mind as well that electrical power fluctuations may alter the wavelength (germicidal UV has a narrow bandwidth).^34^ Lamp cooling by direct airflow reduces UV output. Its effectiveness depends on line-of-sight exposure for UV rays to make direct contact with the organisms.^34^ A further restriction of UV radiation is that it is not sporicidal and HBV can survive exposure.^35^ Dust and other film can coat the UV bulb or fluorescent tube and reduce output.^34^ These limitations of UV preclude its use for sterilisation of medical instruments. In the health-care environment, UV is limited to destruction of airborne organisms or inactivation of microorganisms on surfaces in operating rooms, isolation rooms and biologic safety cabinets.^20^ Chlorhexidine was used by some GPs for items to be sterilised and disinfected. It is not recommended for disinfection of patient care items.^20^ Some GPs used boiling water for sterilisation. Boiling water, however, is not recommended for sterilisation but can be used for high level disinfection, if items are boiled for 20 minutes.^21^ Two GPs used steam autoclaves but did not comply with the recommended guidelines for temperature, holding times and pressures. A sterilisation hold temperature of 134 °C for 3 minutes at a pressure of 225 kPa (2.25 bar) is optimal, or at 121 °C at a pressure of 115 kPa (1.15 bar) for 15–20 minutes.^36^ GPs in other countries also do not fully comply with standards. In a survey in Northern Ireland, 17% of GPs using a steam steriliser used a sub-optimal temperature for sterilisation.^3^ In a UK study, 70% of GPs performed no user check at all on their autoclaves and 15% of instruments were inadequately decontaminated.^5^ Steam sterilisation, that is, moist heat in the form of saturated steam under pressure, is the most dependable method of sterilisation and is the method of choice for reusable instruments in primary care.^20^ Disposable instruments are an alternative to reusable ones. In this study, only a few GPs used disposable instruments.

Almost all GPs separated healthcare (risk) waste from general waste. However, a few inappropriately disposed of risk waste in the municipal domestic waste disposal service. It was reassuring that all GPs, including those working in small rural towns, were able to dispose of sharps by using either a medical waste disposal company or a hospital incinerator.

Various methods were used to clean ultrasound probes and reusable ECG electrodes. After transabdominal ultrasound, it is recommended that the probe be washed with lukewarm water and a mild detergent to remove all residues.^37^ Reusable ECG electrodes should be cleaned in a similar manner.

Fifty per cent of GPs did not have a policy for airflow movement in the surgery and 65% did not triage patients with a cough, into a separate waiting area. Common infections that are transmitted by the airborne route include chickenpox and measles whilst influenza, bacterial pneumonia and meningitis are spread by droplets. Tuberculosis (TB) is spread by both routes. Given the high prevalence of TB in South Africa, policies should be implemented to minimise the risk of nosocomial infection.

Eighty-seven per cent of GPs took responsibility for the washing of soiled linen and 32% used cold water. Whilst there is little evidence that disease is spread by reusable linen, current guidelines recommend washing at a temperature of 65 °C for at least 10 minutes, or ideally at 71 °C for at least 3 minutes to eradicate HIV and hepatitis viruses.^38^

GPs expressed interest in improving their knowledge about infection control to the extent that 80% requested a seminar. In their comments on the questionnaire, four GPs expressed a need for guidelines on infection control. These sentiments imply that there is a need for guidelines to be developed for infection control in general practice in South Africa.

### Limitations

Limitations of the study include a low response rate (34%). However, postal questionnaires in general have a low response rate (< 30%).^39^ It is possible that a telephone survey may have yielded a better response rate, but in a telephone survey of infection control in 92 general practices in the UK, the response rate was only 42%.^5^ Moreover, answers are self-reported so that it is not possible to verify them objectively. The questionnaire was, however, essentially a self-audit, and consequently it is likely that answers reflected actual practice. Finally, it was confined to the Eastern Cape and the results are not necessarily generalizable to the rest of South Africa.

## Conclusion

Overall, GPs are aware of infection control precautions and are interested in further education thereon. Eight-five per cent performed one or more procedures requiring sterilisation or high-level disinfection of medical devices. However, ultraviolet sterilisers and chlorhexidine are not recommended for sterilisation or high level disinfection of medical devices, and their use should be discontinued by GPs. Steam sterilisation is the optimal method for sterilisation and disinfection for most devices in GP surgeries. In addition, hand rubs are underutilised in GP practices. GPs should implement Transmission Based Precautions to prevent airborne and droplet infections.
